# Norovirus in Captive Lion Cub (*Panthera leo*)

**DOI:** 10.3201/eid1307.070268

**Published:** 2007-07

**Authors:** Vito Martella, Marco Campolo, Eleonora Lorusso, Paolo Cavicchio, Michele Camero, Anna L. Bellacicco, Nicola Decaro, Gabriella Elia, Grazia Greco, Marialaura Corrente, Costantina Desario, Serenella Arista, Krisztián Banyai, Marion Koopmans, Canio Buonavoglia

**Affiliations:** * University of Bari, Valenzano, Bari, Italy; † Giardino Zoologico di Pistoia, Pistoia, Italy; ‡ University of Palermo, Palermo, Italy; § Baranya County Institute of State Public Health Service, Pécs, Hungary; ¶ National Institute of Public Health and the Environment, Bilthoven, the Netherlands

**Keywords:** African lion, Panthera leo, calicivirus, norovirus, enteritis, zoonosis, dispatch

## Abstract

African lions (*Panthera leo*) are susceptible to viral diseases of domestic carnivores, including feline calicivirus infection. We report the identification of a novel enteric calicivirus, genetically related to human noroviruses of genogroup IV, in a lion cub that died of severe hemorrhagic enteritis.

Lions (*Panthera leo*) are susceptible to viral diseases of domestic carnivores, including infections with canine distemper virus, feline parvovirus, feline retroviruses, feline herpesvirus, and feline calicivirus (FCV) (*1*–*4*). Antibodies to FCV have been detected in captive lions (*2*), and calicivirus-like particles have been detected in oral vesicular lesions of captive immature lions (*3*). Despite the presence of FCV-specific antibodies and the observation that cub survival may be reduced during calicivirus outbreaks, clear signs of FCV-induced illness have not been described in free-ranging lions (*4*). We detected a novel calicivirus in a 4-week-old lion cub that died of severe hemorrhagic enteritis.

## The Study

In autumn 2004, the Zoo of Pistoia, Italy, adopted 2 adult lions that had been born in captivity. In October 2005 and May 2006, the female gave birth to 2 cubs each delivery, which 3–4 weeks later showed signs of enteritis and died. In October 2006, she gave birth to a single cub, which died of severe hemorrhagic enteritis at 4 weeks of age. The cub exhibited anorexia, depression, and mild dehydration, but it was not moved away from the mother for ethologic and management reasons. In the subsequent days, the cub’s general condition appeared to worsen; anorexia and more marked depression were reported by the animal caretakers. Therefore, on day 3 after illness onset, the cub was taken to the zoo’s animal hospital. Examination showed a temperature of 38.6°C, hemorrhagic enteritis, tenesmus, and deep sensorial depression. Hydration and antimicrobial therapy were immediately started, but after 24 hours the animal was agonal and hypothermic and was therefore euthanized. At necropsy, severe hemorrhagic enteritis, hemorrhage in the intestinal lymph nodes, and marked dehydration were observed. Histologic examination showed marked alteration of the intestinal mucosa: erosions, villi depletion, and hemorrhagic infiltration.

The tissues and intestinal contents were screened for common feline and canine viral pathogens by using either conventional or quantitative PCR and reverse transcription–PCR (RT-PCR). Results were negative for known feline (parvovirus, coronaviruses, herpesvirus, retroviruses) and canine (distemper virus, parvovirus, adenoviruses type-1 and type-2) pathogens. Calicivirus was identified in the intestinal content by using a broadly reactive primer pair, p289-p290, targeted to highly conserved motives of the RdRp region of the polymerase complex (*5*), but unexpectedly, the strain could not be characterized as FCV by using multiple sets of primers specific for the FCV capsid gene. In addition, the sample was positive for the norovirus (NoV)-specific primer pair JV12Y-JV13I (*6*).

Bacteriologic investigations detected an *Esherichia coli* O86, enteropathogenic *E. coli* (EPEC) group. *Clostridium sordelli* and *C. perfringens* were also isolated. By screening of the *cpb, cpb2, etx,* and *cpe* genes, the *C. perfringens* isolate was characterized as toxin-type A.

**Figure 1:**
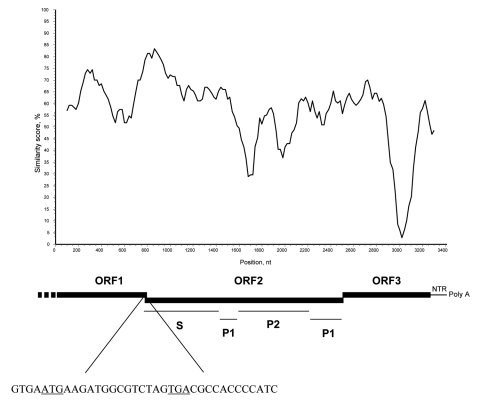
Genome organization of the lion norovirus (NoV) 387/06. A nucleotide identity plot of the genome of the lion NoV (from the 3′ end of open reading frame [ORF] 1 to the poly-A tail) was compared with the human genogroup IV.1 NoV, Fort Lauderdale/560/98/US (AF414426). The sequences were analyzed with Simplot software (http://sray.med.som.jhmi.edu/scroftware/simplot) by using a window size of 200 and step size of 20 with gap strip off and J-C correction on. The ORF1–ORF2 junction region is shown with the starting and stopping codons ATG and TGA underlined. The highly conserved domain S and the highly variable domains P1 and P2 of the capsid protein are also indicated.

**Figure 2:**
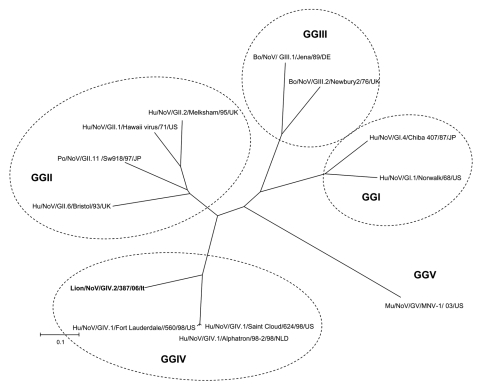
Phylogenetic tree constructed on the full-length amino acid (aa) sequence of the capsid protein. The tree was constructed by using a selection of norovirus (NoV) strains representative of genogroups (GG) I to V. Phylogenetic reconstruction was carried out with the p-distance correction and the neighbor-joining method, supported with bootstrapping >1,000 replications. Distance analysis and phylogenetic inference were carried out using the Mega 3.0 software package (www.megasoftware.net). Strain classification follows the outlines of Wang et al. (*7*) and Zheng et al. (*8*); strain designation follows the outlines of Green et al. (*9*). Bo, bovine; DE, Germany; UK, United Kingdom; Hu, human; JP, Japan; US, United States; Mu, murine; NLD, the Netherlands; Po, porcine.

Sequence analysis of the 315-bp fragment of the RdRp region (strain 387/06) by using BLAST (www.ncbi.nlm.nih.gov/blast) and FASTA (www.ebi.ac.uk/fasta33) showed that the virus was distantly related to FCV (<35% amino acid [aa] identity) but closely related to human and animal NoVs (≤ 75% aa identity). To determine the sequence and genome organization of the novel calicivirus, a 3.4-kb region at the 3′ end of the genome was amplified by RT-PCR as described by Wang et al. (*7*). The sequence of the 3′ end of open reading frame (ORF)1, the full-length ORF2, ORF3, and the noncoding region through the poly-A tail was determined (GenBank accession no. EF450827). A 14-nt overlap was present in the ORF1–ORF2 junction region, as it is in most human and animal NoVs. The ORF2 was 1,737 nt long and contained an ORF encoding a capsid protein with a predicted size of 578 aa. By BLAST and FASTA analysis, the highest sequence match was found to genogroup IV NoVs (69.3–70.1% aa identity), and identity to non-GIV NoVs was ≤52.6% aa. A total of 23 aa insertions, scattered throughout the P2 domain, were present in the capsid protein of the lion NoV when compared with human genogroup IV NoVs. A 1-nt overlap was found between ORF2 and ORF3, and a 106-nt long nontranslated region was found between ORF3 and the poly-A tail. ORF3 was 765 nt long and encoded for a 254-aa polypeptide. The nucleotide identity plot of the genome of the lion NoV (from the 3′ end of ORF1 to the poly-A tail) was compared with the human genogroup IV.1 NoV, Fort Lauderdale/560/98/US (AF414426) (Figure 1). A phylogenic tree was constructed by using the capsid protein of a selection of human and animal NoVs of the various NoV genogroups (I to V) (*7*,*8*). In the tree (Figure 2), the lion calicivirus strain was grouped with genogroup IV human NoVs.

## Conclusions

NoVs in humans were first discovered by use of electron microscopy in 1972 (*10*). As a consequence of the development and large-scale application of new and sensitive molecular diagnostic techniques, NoVs are now regarded as the major cause of epidemic, nonbacterial gastroenteritis worldwide in humans of all age groups (*9*). Human NoVs are classified into genogroups I, II, and IV. In addition, NoVs classified in genogroups II and III have been detected in pigs and cows (*7*,*11*,*12*), and NoVs proposed as genogroup V have been detected in mice (*13*) (Table). However, to our knowledge, NoVs have not been detected in other animal species and our report is the first description of NoVs in felids.

**Table:** Distribution of norovirus genogroups and genotypes

Host	Genogroup and genotypes*				
	I	II	III	IV	V
Human	1–8	1–10, 12–17		1	
Pig		11, 18, 19			
Cattle			1, 2		
Lion				2†	
Mouse					1

*Norovirus classification follows the outlines of Wang et al. (*7*) and Zheng et al. (*8*). †Determined in this study.

Because of the possibility of genetic recombination, a consistent and reliable classification of NoV is necessarily based on analysis of the complete capsid gene, and a comprehensive classification scheme has been established by analysis of 164 NoV strains (*8*). Strains within the same genotype (or cluster) share >85% aa identity; strains of different genotypes within the same genogroup share 55%–85% aa identity (*8*). The lion NoV 387/06 appeared to be more related genetically to human genogroup IV NoVs (69.3%–70.1% aa identity in the capsid protein). Accordingly, the virus may be considered as a distinct genotype (IV.2) within genogroup IV; human genogroup IV NoVs are genotype IV.1.

The close genetic relationship observed between the lion NoV strain and human genogroup IV NoVs reinforces the notion that the evolution of human NoVs is intermingled with that of animal NoVs. The mechanisms driving the evolution of NoVs are accumulation of punctuated mutations and recombination (*14*). In addition, NoVs can infect heterologous species, resulting in mild or unapparent infections (*15*). To assess whether animal NoVs have emerged over time in humans by direct interspecies transmission or by exchange of genetic material through recombination with human NoVs, the genetic diversity of animal NoVs must be explored.

To acquire epidemiologic information, either single or pooled fecal samples of overtly healthy animals from the zoo were screened by RT-PCR with broadly reactive or specific primer sets. Samples of adult and immature lions, tigers (*P. tigris*), jaguars (*P. onca*), manul cats (*Otocolobus manul)*, siberian lynxes (*Lynx lynx wrangeli*), fennecs (*Vulpes zerda*), polar bears (*Ursus maritimus*), and wolves (*Canis lupus*) were screened; calicivirus RNA was not detected.

Whether the novel lion calicivirus is a newly identified felid viral pathogen or a NoV strain of heterologous origin detected incidentally in the intestinal content of the cub remains to be proven. Bacterial coinfections were also detected and likely enhanced the severity of the enteritis disease by triggering synergistic effects. Accordingly, the pathogenic potential and the origin of the novel calicivirus strain remain to be elucidated.
